# Temporal properties of inferior colliculus neurons to photonic stimulation in the cochlea

**DOI:** 10.14814/phy2.12491

**Published:** 2015-08-26

**Authors:** Xiaodong Tan, Hunter Young, Agnella Izzo Matic, Whitney Zirkle, Suhrud Rajguru, Claus-Peter Richter

**Affiliations:** 1 Department of Otolaryngology, Feinberg School of Medicine, Northwestern UniversityChicago, Illinois, USA; 2 Department of Biomedical Engineering, University of MiamiMiami, Florida, USA; 3 Department of Otolaryngology, University of MiamiMiami, Florida, USA; 4 Department of Biomedical Engineering, Northwestern UniversityEvanston, Illinois, USA; 5 The Hugh Knowles Center, Department of Communication Sciences and Disorders, Northwestern UniversityEvanston, Illinois, USA

**Keywords:** Infrared neural stimulation, limiting rate, single unit

## Abstract

Infrared neural stimulation (INS) may be beneficial in auditory prostheses because of its spatially selective activation of spiral ganglion neurons. However, the response properties of single auditory neurons to INS and the possible contributions of its optoacoustic effects are yet to be examined. In this study, the temporal properties of auditory neurons in the central nucleus of the inferior colliculus (ICC) of guinea pigs in response to INS were characterized. Spatial selectivity of INS was observed along the tonotopically organized ICC. Trains of laser pulses and trains of acoustic clicks were used to evoke single unit responses in ICC of normal hearing animals. In response to INS, ICC neurons showed lower limiting rates, longer latencies, and lower firing efficiencies. In deaf animals, ICC neurons could still be stimulated by INS while unresponsive to acoustic stimulation. The site and spatial selectivity of INS both likely shaped the temporal properties of ICC neurons.

## Introduction

The electrode–tissue interface constitutes one bottleneck for neural prostheses. Electrical current is delivered from the contacts of the interface to stimulate the neurons. Determined by the electrical properties of the tissue, the current spreads widely in the tissue and overlapping electrical current fields from different channels result in a broad activation of neurons. The high fidelity of the pristine neural system cannot be restored. In particular, in cochlear implants current spread results in the stimulation of large populations of spiral ganglion neurons (SGN) along the cochlea (Ryan et al. 1990; Suesserman and Spelman 1993; McKay et al. 1995; Jolly et al. 1996; Matsushima et al. 1997; Kral et al. 1998; Liang et al. 1999; Cohen et al. 2004; Mens and Berenstein 2005; Micco and Richter 2006; van den Honert and Kelsall 2007; Landsberger and Srinivasan 2009; Bierer et al. 2010; Srinivasan et al. 2010; Landsberger et al. 2012; Bingabr et al. 2014). Multipolar stimulation (Smith et al. 2013; George et al. 2014; Kalkman et al. 2014), current steering (Koch et al. 2007; Anderson 2008; Berenstein et al. 2008; Bonham and Litvak 2008; Frijns et al. 2009; Srinivasan et al. 2010; Donaldson et al. 2011; Wu and Luo 2013; Dumm et al. 2014), or improved cochlear implant electrodes have been employed to enhance the performance of the implant users. Among the efforts to increase the selectivity of SGNs stimulation, optical methods such as optogenetics (Hernandez et al. 2014a,b) and the use of pulsed infrared lasers has been proposed (for a review see Richter and Tan 2014; Izzo et al. 2006). Optical methods have the potential to stimulate discrete neuron populations and thus increase the number of independent channels that can be used at the neural interface to encode acoustical information (Richter et al. 2011). While optogenetics works through the activation of a photosensitive ion channel, which was expressed in the cell membrane of the neuron, INS works through local heating of the target area and requires the heat delivered with each pulse to dissipate (Wells et al. 2007; Suh et al. 2009; Shapiro et al. 2012; Thompson et al. 2013a,b). While stimulation with light potentially provides a larger number of independent channels, the rate by which pulses can be delivered is limited. For optogenetics, the rate is limited by the kinetics and the photon efficiency of the optogenetic tool; for INS, the rate is limited by the dissipation of the heat deposited with each pulse. At present, the maximum usable rate of stimulation in the cochlea was ∼70 Hz for the optogenetic approach (Hernandez et al. 2014a) and ∼200 for INS (Goyal et al. 2012; Matic et al. 2013). The question is whether this is fast enough.

It has been published previously that for cochlear implant users the performance in speech perception tests is correlated with the ability to detect modulations in an acoustic stimulus. The better modulations are detected the better the performance in the speech tests (Cazals et al. 1994; Fu 2002). This finding indicates that stimulus conditions that interfere with temporal aspects required for encoding modulation will restrict the information about temporal aspects to the cochlear implant user. Hence, the carrier frequency may play an important role in processing speech with a cochlear implant. Today, speech processors typically use stimulation rates of 250 (SPEAK processing strategy) to 2400 pulses per second (pps). Although it has been argued that pulse repetition rates above 2000 pps evoke more natural (stochastic) firing pattern of single auditory nerve fibers and better transmit temporal information (Wilson 1997; Rubinstein et al. 1999), systematic studies have not consistently confirmed the results (Brill et al. 1997; Fu and Shannon 2000; Galvin and Fu 2005; Arora et al. 2009, 2011; di Lella et al. 2010; Shannon et al. 2011; Duran et al. 2012; Park et al. 2012).

Stimulation rates above 200 Hz, however, are a challenge for optical stimulation. For optogenetics, the rate is at present limited by either the speed or the efficiency of the optogenetic tool (Bernstein and Boyden 2011; Grossman et al. 2011; Yizhar et al. 2011). Since INS requires the temporal and local heating of the target structure, a net temperature increase may occur with increasing pulse repetition rates. The heat balance in the tissue, heat delivery, and heat diffusion will therefore determine the maximum repetition rates. In a long-term study it has been shown that safe INS is possible at a 200 Hz pulse repetition rate with 12 *μ*J/pulse radiant energy and 100 *μ*s pulse length (Matic et al. 2013), without damaging the cochlea. Nevertheless, recordings from single auditory nerve fibers have demonstrated that most of the neural responses cannot follow a stimulation rate faster than 97 Hz (Littlefield et al. 2010). The authors have pointed out that the neuron population presented might have been biased by the selection process during the experiments and that repetition rates at higher frequencies are possible. For electrical stimulation, sustained rates obtained from the auditory nerve adapted over a short period of time. Electrical rate–intensity curves are steep, often exceeding 200 spikes/sec within 6 dB of threshold (Hartmann et al. 1984; Javel 1996). In other words, typically sustained rates were just above 200 Hz. This is faster than what has been reported for acoustical stimulation. Acoustic rate–intensity curves for single auditory nerve fibers generally saturate at or below 200 spikes/sec (Gifford and Guinan 1983). Higher response repetition rates during electrical stimulation of the cochlea were recently reported for the central nucleus of the inferior colliculus (ICC), which was as high as 600 Hz (Middlebrooks and Snyder 2010).

We are interested in how the temporal pattern in the ICC compares for INS. In this study, we examined the temporal properties of auditory neurons in the ICC to INS and acoustic stimuli. Measurements were made in normal hearing, acutely deafened, and chronically deaf animals. Single tungsten electrodes and 16-channel electrode arrays were used for the measurements. The experiments were performed to characterize and compare the response pattern of units in the central nucleus of the inferior colliculus to trains of acoustic clicks and to trains of laser pulses. Significant differences between the response properties of ICC single units to INS and acoustic stimulation were observed. These results also contribute to the dispute of the mechanism of INS by providing evidence of direct stimulation of SGNs in the cochlea.

## Materials and Methods

Experiments were conducted in normal hearing (*N *=* *18), acutely deafened (*N *=* *4), and chronically deaf (*N *=* *4) guinea pigs of either sex weighing 200–600 g. All procedures were carried out in accordance with the NIH Guide for the Care and Use of Laboratory Animals and were approved by the Institutional Animal Care and Use Committee at Northwestern University.

### Animal surgery and electrode placement

#### Animal anesthesia and animal monitoring

Anesthesia was induced by an intraperitoneal injection (i.p.) of urethane (0.9 mg/kg) in a 20% Ringer’s Lactate (RL) solution. At 15 min intervals, the level of anesthesia was assessed by a paw withdraw reflex. Supplemental doses of ketamine (40 mg/kg) and xylazine (2.5 mg/kg) were given with RL solution when required. The body temperature of the animals was maintained at 38°C with a heating blanket. A BM3-Vet system (Bionet Co. Ltd, Seoul, Korea) served to continuously monitor the animal’s vital signs, including heart and respiratory rates and blood oxygen saturation.

#### Surgical access to the cochlea

Surgical procedures were similar to those previously described (Richter et al. 2011; Tan et al. 2015a). The heads of the animals were mounted in a stereotactic head holder (Stoelting, Kiel, WI) by fixing the palate and teeth with a bite bar and the ears with ear bars. Next, the frontal bony skull was surgically exposed and 2–3 bone screws were anchored into the skull. A metal rod, which was attached to the stereotactic frame, was lowered between the screws on the head and was fixed to the animal’s head with dental acrylic (Methyl methacrylate, Co-oral-ite Dental MFG Co., CA). After the dental acrylic cured, the left ear bar was removed and a c-shaped skin incision was made behind the left pinna. The cervicoauricular muscles were removed by blunt dissection and the cartilaginous outer ear canal was exposed and cut. The left bulla was exposed and opened approximately 2 × 3 mm with a motorized drill (World Precision Instruments, Sarasota, FL). The basal turn of the cochlea was identified and a cochleostomy was created with a 0.5-mm Buckingham footplate hand drill (Richards Manufacturing Co., Memphis, TN) approximately 0.5 mm apical to the bony rim of the round window.

After the cochleostomy was made in the basal cochlear turn, an optical fiber (P200-5-VIS-NIR, Ocean Optics, Dunedin, FL) was inserted through the opening of the cochlear wall. For the present experiments, the fiber was 200 *μ*m in core diameter, with a numerical aperture of 0.22 and an acceptance angle of 25.4° in air. The optical fiber was mounted to a micromanipulator (MHW103, Narishige, Tokyo, Japan) to ensure consistent orientation during stimulation. To measure compound action potentials (CAPs), a silver ball electrode was placed on the round window.

#### Access to the ICC and placing the recording electrode

**Figure 1:**
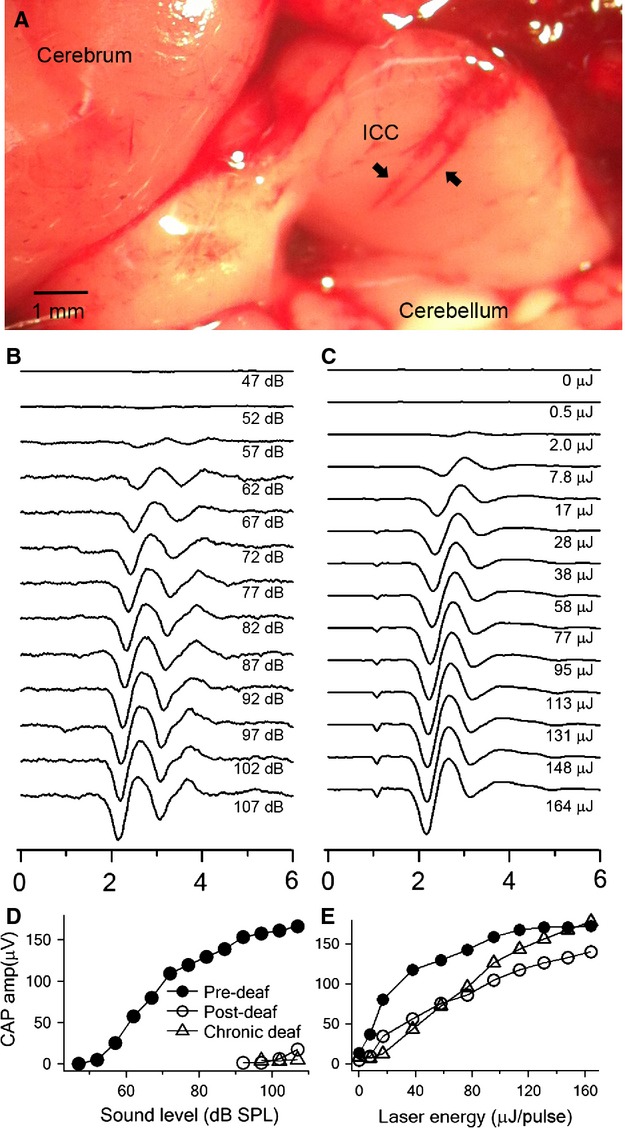
Verification of positioning in ICC and animal hearing with Compound action potentials (CAP). (A) Dissection picture of the brain showing the ICC and the tracks of tungsten electrode. The structures of the brain are indicated and two tracks of tungsten electrode penetrations are shown (the arrows). (B) Typical CAP waveforms induced by acoustic clicks. The numbers on the right indicate the corresponding sound level. Each waveform is the averaged response of 50 repeated stimulations. (C) CAP waveforms induced by INS from the same animal. The numbers on the right indicate the corresponding energy level. (D) Acoustic CAP (aCAP) amplitude-level curves from pre-, post-deafening and chronically deaf animals. The pre-deaf curve (solid circles) was derived from the CAP traces in panel A. (E) Optical CAP (oCAP) amplitude-level curves from pre-, post-deafening and chronically deaf animals. The pre-deafening curve (solid circles) was derived from the CAP traces in panel B. Note that the post-deafening aCAP threshold shift was over 40 dB SPL. Panels B, C and the circles in D and E were all from the same animal.

As previously described in detail, the ICC was surgically accessed to place either a tungsten electrode (TM31A10, 1 MΩ. WPI, Sarasota, FL) or a multichannel electrode array (A1x16-5 mm-100-177, NeuroNexus Technologies, Ann Arbor, MI) to record neural responses from single units (Richter et al. 2011). The right temporalis muscle was reflected, and an approximate 5 × 5 mm opening was made in the right parietal bone just dorsal to the temporoparietal suture and just rostral to the tentorium. A small incision in the dura mater was then made allowing for placement and insertion of the electrodes. Single tungsten electrodes were mounted on a microdrive (Burleigh 6000 ULN controller, Burleigh Instruments, NY), which was placed on a 3D coarse manipulator. Thus, stereotactic placement of the electrode was possible. The electrodes were connected to a differential amplifier (ISO-80, WPI, Sarasota, FL) with a high input impedance (>10^12^ Ω) and a gain of 60 dB to record neural activity from identified single units. The electrode was stepped into the ICC also at a 45° trajectory using the microdrive. Neural responses evoked by acoustic or laser stimulation were monitored and recorded. The advancement of the electrode was recorded relative to surface of the cortex. After the experiments, the recording sites were verified visually (Fig.1A). Using midcortical sections, the thickness of the visual cortex overlaying the ICC was measured. The average value of 3.47 mm (*n *=* *6) was subtracted from the recorded position to provide the penetration depth of the electrode into the inferior colliculus. To obtain precise depth–frequency mapping of ICC, the cortex was removed in some experiments and the advancement of the electrode into ICC was directly visible. This depth–frequency mapping was later used to normalize the depth of each recording sites along the multichannel electrode array (see Results section).

Multichannel electrodes were also used for ICC recordings in normal hearing animals. The silicon substrate, thin film, multichannel penetrating electrode array had 16 recording sites (177 *μ*m^2^/site) along a 5.0 mm shank at center-to-center intervals of 100 *μ*m. The multichannel electrode arrays were attached to the stereotactic head holder and then advanced through the occipital cortex into the ICC using a 3D micromanipulator (Stoelting, Kiel, WI) along a dorsolateral to ventromedial trajectory at approximately 45° off the parasagittal plane. Using this trajectory, the electrode array passed through the central nucleus of the ICC approximately orthogonal to its isofrequency laminae (Snyder et al. 2004, 2008; Richter et al. 2011). After the initial placement of the distal tip of the electrode into the ICC, the electrode was advanced, while an acoustic tone pip was presented to the left ear. When neural responses to a tone pip between 16 and 25 kHz could be evoked from the distal contact of the array, the electrode was placed correctly. In some instances, the electrode was advanced several times into the ICC before the desired placement was achieved.

#### Acute and chronic deafening

Chronically deaf animals received a transtympanic injection of neomycin (25 mmol/L, ∼250 *μ*L in RL solution) at least 4 weeks prior to the experiments. Acute deafening was achieved by injecting 25 *μ*L of neomycin (25–30 mmol/L) into scala tympani through the cochleostomy. In each case, cochlear function was assessed by measuring CAP thresholds.

### Stimulation of the cochlea

#### Acoustic

Voltage commands for the acoustic stimuli were generated with a computer I/O board (KPCI 3110, Keithley, Cleveland, OH) integrated into a PC and were used to drive a Beyer DT 770Pro headphone (Beyerdynamic, Farmingdale, NY). The speaker’s speculum was placed directly in front of the cartilaginous outer ear canal after the pinna was removed. The acoustic output was calibrated with a 1/8-inch microphone (Brüel & Kjær North America Inc., Norcross, GA). To monitor cochlear function, acoustic condensation clicks (50 *μ*sec) and tone pips were used. Tone pips were 12 msec long, including a 1-msec rise/fall, with different carrier frequencies between 0.5 and 32 kHz, and were presented at sound levels between 0 and 110 dB sound pressure level (SPL, re 20 *μ*Pa). Tone pips were presented at a rate of ∼4 Hz. After a single neural unit was identified, its characteristic frequency (CF) was determined. Acoustic clicks were presented at 2, 10, 20, 40, 80, 120, 200, 300, and 500 Hz and at different sound levels (0–107 dB SPL at steps of 5 dB).

#### Optical

Optical stimulation was achieved with a diode laser (Lockheed Martin Aculight Corp., Bothell, WA). For the present experiments, the wavelength was selected at 1855 nm and the pulse duration at 60 or 100 *μ*sec. The laser operated at 2, 10, 20, 40, 80, 120, 200, 300, or 500 Hz repetition rate and was coupled to the optical fiber. Previously we have measured the full width half maximum (FWHM) value for the optical spot at the tip of the optical fiber. It was 130 *μ*m in diameter, with a Gaussian energy distribution as determined by using the knife-edge technique (Teudt et al. 2007; Richter et al. 2011). The radiant energy per pulse at the tip of the optical fiber was measured in air with the J50LP-1A energy sensor (Coherent, Santa Clara, CA) and was 0–164 *μ*J/pulse. The penetration depth of the radiation at 1855 nm is about 771 *μ*m, assuming primarily water absorption (e.g., Hale and Querry 1973). Based on previous experiments, distances between the tip of the optical fiber and the SGN were between 200 and 500 *μ*m (Moreno et al. 2011). Since the incident energy decreases in water by 1/e for each 771 *μ*m traveled along the optical path, it is a fair assumption that the energy at the SGN is about one-third of the energy measured at the tip of the optical fiber.

### Variables measured

#### CAPs

compound action potential responses to acoustic clicks were measured and used to monitor cochlear function regularly during the experiments. The condensation clicks were delivered via the speaker and the electrical responses were recorded with a round window electrode. CAP responses were also recorded with pulsed INS (100 *μ*sec) delivered at 10 Hz. The electrode was also connected to an ISO-80 differential amplifier. The band-pass filter settings were 0.3–3 kHz and the sampling rate was 250 kHz. The noise in the recordings was typically about 10 *μ*V.

#### Action potentials of ICC single units recorded with tungsten electrode

**Figure 2:**
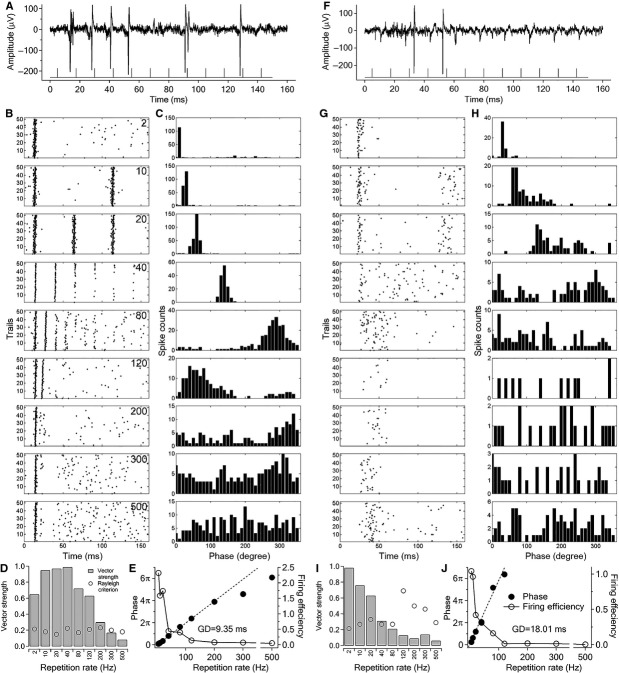
Examples of ICC single unit responses to INS at laser radiant energy of 136 *μ*J/pulse. Pulse trains were presented at about 2 Hz. ([A–E] unit 030413_N02; [F–J] unit 052913_N01) (A) Single traces of evoked action potentials generated by repeated stimulation at 80 Hz. The line with spikes at the bottom of the plot represents the INS train. (B) Raster plots of single unit firing to 50 repeated stimulations in a 160 msec interval. Each row of panels represents responses to one stimulus repetition rate which is indicated by the number in the upper right corner (Unit: Hz). Each dot in the panels represents one action potential as shown in A and each row in the panels represents the responses to one stimulation train. A total of 50 trials in each panel represent 50 repeated stimulations. (C) Period histogram (phase) plots of the same responses in panel B. Note the phase-locked firing at low frequencies. The mean phase represents the averaged latency or group delay of the responses in the stimulation cycle. (D) Vector strengths at different repetition rates for the responses in panel B and C, and related Rayleigh criteria for statistical significance level of *P* < 0.001. Note the highest phase-locked firing rate, or the limiting rate is 200 Hz. (E) Left axis and the black dots: mean phase plotted against the stimulation repetition rate of the response in panel C. Note the mean phase has a linear relationship with the repetition rate up to its limiting rate (200 Hz). The slope of the linear curve fitting gives the group delay which was 9.35. Right axis and the circles: firing efficiency at different repetition rates calculated from the raster plot in B. (F–J) Same plots as (A–E) for single unit 052913_N01.

Neural activities in the ICC were monitored with an oscilloscope and recorded continuously through a KPCI 3110 I/O board during the stimulations. The action potentials were distinguished from field potentials in that the amplitude of the spikes did not change with an increase in the stimulation amplitude (see examples in Fig.2A and F). The action potentials at least twice the noise level (usually about 80 *μ*V) and with uniform waveforms were isolated as single unit responses (see examples in Fig.2A and F). For multiunit responses, different characteristics of the action potentials would have been expected as we have described recently (Tan et al. 2015b).

#### Measurements with multichannel electrode arrays

Data were acquired simultaneously using the 16-channel Plexon system (Plexon Inc., Dallas, TX). Using the SortClient software, the trigger threshold for each channel was determined manually and was set individually according to the noise and response levels of each channel. The Plexon system records and stores the time points and snippets of the waveforms of the action potentials. Each channel had a 40 kHz sampling rate, with a 16-bit analog/digital input conversion and a 250 Hz low pass filter.

From our previous experiments, we are aware that the multichannel electrode tended to pick up more neural activity, which likely comes from indistinguishable multiple units (Tan et al. 2015b). Based on its limitations, the multichannel electrode recordings were mostly used to record in acutely deafened animals and for pre- and postdeafening comparisons. The trigger threshold for data acquisition was also set at least twice the noise level, and any ambiguous spikes with variable waveforms were excluded from analysis.

### Data analysis

The initial multichannel recordings were resorted with the Offline Sorter software (Plexon Inc.). Single or multiple units were identified according to their amplitudes, shapes, and response types. The resorted files were transferred to MATLAB (MathWorks Inc., Natick, CA) for further analysis. Single tungsten electrode recordings were also analyzed in MATLAB. Custom-written software was used to generate raster plots, spike counts and latencies, poststimulus time histograms (PSTH), and interstimulus interval (ISI) histograms.

Spike rate (SR) at each energy level and the stimulus repetition rate were determined. Firing efficiency (FE) was calculated using the following equation: FE = (SR − SR_spon_)/R_stim_, where SR is the number of spikes per stimulation train, SR_spon_ is the SR at energy level 0, and R_stim_ was the number of pulses in each stimulation train.

The phase lock of the firing was evaluated by calculating the vector strength. It is generally used for evaluation of circular uniformity and widely adopted as a way to assess the strength of phase-locked responses of auditory neurons (Goldberg and Brown 1969; Littlefield et al. 2010; Middlebrooks and Snyder 2010). The time period between two stimulation pulses was defined as the cycle time. Each spike was assigned into the circle as a vector with unit length and phase angle *α*, as calculated by following equation:



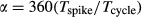


where *T*_spike_ is the time between the stimulation pulse and the evoked spike and *T*_cycle_ is the interval between two stimulation pulses determined by repetition rate. The *x* and *y* components of the vector were then calculated as: *x *= cos(*α*) and *y *= sin(*α*). The averaged phase *θ* was given by 50 summed trials determined by following equation:



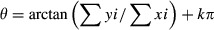


where *k* equals zero or one depending on the signs of Σ*x*i and Σ*y*i. The vector strength was given by the length of mean vector *r* calculated by following equation:



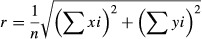


The vector strength varied between 0 and 1, and the bigger the value, the stronger the phase locking.

Rayleigh’s test for circular uniformity (Mardia 1972) was used to test for statistical significance at a level of *P *<* *0.001. The highest repetition rate at which the vector strength was higher than the Rayleigh criterion was defined as the limiting rate of the unit. ICC units tended to have onset responses to pulse trains at high repetition rates, which might generate a misleading phase-locked result. Therefore, the limiting rate was determined by the vector strength calculated from the spikes 50 msec after the onset of the stimulus train.

## Results

### Acoustic- and laser-evoked CAPs

The CAP was used for an initial assessment of the cochlear function and for the evaluation of the cochlear damage, which occurred during the progression of the study. Figure1 compares CAPs evoked by acoustic clicks (aCAP) and optical pulses (oCAP) in normal hearing, acutely deafened, and chronically deaf animals. Figure1B and C, and circles in 1D and 1E were all from the same animal. Figure1B and C show aCAP and oCAP traces from the animal predeafening. The maximum peak-to-peak amplitudes were both around 160 *μ*V. The threshold was 52 dB SPL for aCAP (Fig.1B and the circles in Fig.1D) and 2.0 *μ*J/pulse for oCAP (Fig.1C and the circles in Fig.1E). After acute deafening, the animal showed a significant elevation in aCAP threshold (102 dB SPL, Fig.1D, the circles). In contrast, the oCAP did not change drastically after acute deafening (7.8 *μ*J/pulse, Fig.1E, the circles). The peak amplitude of the oCAP decreased by about 20%, which was consistent with the previous report (Rajguru et al. 2010). Figure1D and E (the triangles) also show an example for a chronically deaf animal (the triangles). For this animal, the aCAP threshold was about 107 dB SPL (Fig.1D), but the oCAP threshold (17 *μ*J/pulse) and maximum amplitude was close to that of normal hearing guinea pigs (Fig.1E).

The CAP varies in both threshold and amplitude among different animals, and varies in postdeafening animals as well. In our study, the click aCAP threshold elevation after acute deafening could vary from 0 to 70 dB SPL, similar to the variations indicated in other studies (Tan et al. 2015a). Therefore, the loss of cochlear function was determined prior to the characterization of the neuronal responses. In the present study, an animal was considered to be chronically deaf when the CAP threshold to acoustic clicks was over 85 dB SPL 1 month after neomycin application. With this criterion, four of the nine treated animals were defined as chronically deaf and were used in the study. For acute deafening, the criterion was a CAP threshold elevation of more than 40 dB at 15–45 min after neomycin treatment. Eight of the 10 animals reached the criterion. INS evoked an oCAP in most animals, including normal hearing, acutely deafened, and chronically deaf ones. Typically, the amplitude of the CAP decreased continuously after cochlear delivery of neomycin. In some acutely deafened animals (four of the eight), stable CAP amplitudes established postdeafening. Only the single units recorded during this stable time period were considered for analysis.

### Temporal properties of ICC single units in response to INS in deaf animals

In most animals, INS evoked neural responses in neurons recorded from the ICC, independent of whether the animals were hearing or deaf. Figure2 shows data from two well-isolated ICC single units (030413_N02 and 052913_N01) from chronically deaf animals recorded with single tungsten electrodes. Both neurons only responded to INS but not to acoustic stimulation at any frequencies. In unit 030413_N02, most spikes evoked by trains of laser pulses, which were presented with a pulse repetition rate of 80 Hz, were at the same time window (or phase) in the stimulation cycle, although the spikes did not follow each stimulation pulse (Fig.2A). The neuron’s response pattern was considered phase-locked firing and was obvious at frequencies lower than 80 Hz as shown in the raster plots (Fig.2B). At higher pulse repetition rates (over 200 Hz), the firing pattern showed short lasting inhibition after the initial response (Fig.2B). The phase of each spike was plotted in the period histograms (Fig.2C), from which the related vector strength was calculated and tested for significance using the Rayleigh criterion as plotted in Figure2D. The vector strength at each repetition rate was higher than the Rayleigh criterion up until 200 Hz. Therefore, the maximum following rate or the limiting rate of the single unit is 200 Hz. The mean phase showed a linear relationship with stimulation frequency at repetition rates up to 200 Hz as well (Fig.2E, the circles and left axis). The slope of the fitted curve gives the group delay (9.35 msec), which is the average latency of all the phase-locked firing. The group delay is slightly longer than the mean first spike latency (FSL) at 2 Hz, 7.70 ± 0.79 msec, but close to that at 40 Hz, 9.38 ± 0.36 msec. The firing efficiency was calculated for each repetition rate, as shown in Figure2E (the circles and right axis). The selected unit had a FE as high as 2.3 at 2 Hz (each pulse induced an average of 2.3 spikes) but decreased rapidly to less than 0.1 at repetition rates over 200 Hz.

Figure2F–J shows another single unit (052913_N01, depth 435 *μ*m) responding to INS. Unlike the previous one, this single unit had no phase-locked firing at 80 Hz as shown in Figure2F. Raster plots showed that the first spike latency was much longer (18.85 ± 1.91 msec at 2 Hz) and more scattered at 2–40 Hz (Fig.2G), which also accounted for the dispersion in the phase plot (Fig.2H). At repetition rates higher than 40 Hz, the onset firing became obscure and the first spikes in each trail scattered in a broad range. At 120 and 200 Hz, the responses were largely inhibited with only a few spikes recorded. The latency was much longer and the jitter much bigger at higher repetition rates (42.31 ± 10.02 and 40.48 ± 8.92 Hz, respectively). For this example, the highest repetition rate with vector strength over the Rayleigh criterion was 40 Hz, and the group latency, generated by the phase plot, was 18.01 msec (Fig.4I). This is close to the FSL at lower repetition rates but much shorter than those at high repetition rates. The unit had a FE of about 1 spike per pulse at low repetition rates that sharply dropped to 0.1 spike per pulse at over 80 Hz (Fig.2J).

Limiting rate determines the maximum frequency of repeating stimulations the SGN can follow, and group delay determines how fast the coded signal can be transmitted. Obviously they are both key temporal properties of ICC single units in response to INS pulse trains and are thus used for representing the temporal properties of ICC neurons in this study.

### Spatial selectivity and acoustic independent of INS responses in ICC

**Figure 3:**
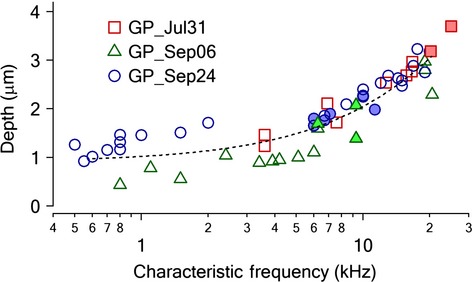
Depth-CF correlation of ICC single units recorded from three animals. Hollow dots indicate single units responded to acoustic stimulation only. Solid dots indicate single units responded both to INS and acoustic stimulation. The dashed line is the curve fitting with the adjusted Greenwood function (see text).



**Figure 4:**
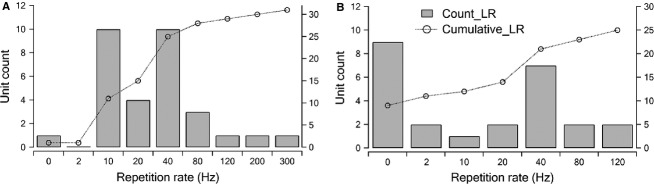
Counts of single units with different limiting rate during INS (bars and the left axis) and the corresponding cumulative distribution (the line with markers and the right axis) recorded with tungsten electrode (A) and multi-channel electrode (B), respectively. LR, limiting rate.

The location of the cochleostomy was always at the basal cochlear turn. Along the tonotopic map of the cochlea, this site corresponds to high frequencies, 6–25 kHz. To determine the precise frequency range for which stimulation was possible with the laser, in both the cochlea and the ICC, in the first set of experiments, the depth–frequency relationship along the ICC was determined in normal hearing animals. A tungsten electrode was inserted along several tracks through the ICC starting at the surface of the ICC. Single unit activity in response to acoustic and/or INS was recorded. Frequencies for pure tones were between 2 and 30 kHz and stimulus levels were between 20 and 110 dB SPL. Threshold for stimulation was determined as the sound level for which the firing rate of the identified unit increased by 20% above the unit’s spontaneous firing rate. The plot of sound levels to reach stimulation threshold versus stimulus frequency resulted in a trace with a single minimum. This plot is also called frequency tuning curve. The frequency at the minimum is defined as the characteristic frequency (CF) for the unit. Figure3 shows the CFs of the single units recorded from the ICC of three different animals. The depth of the recordings ranged from 0.4 to 4.1 mm and the CFs range from 0.5 to 25 kHz. All units (*N *=* *55), obtained from three animals, showed a similar depth–CF relationship in the ICC. The Greenwood function, which was used previously to correlate cochlear position to CF (Greenwood 1961, 1990; Merzenich and Reid 1974), was adapted for the inferior colliculus to correlate distance into the ICC and CF. Data were fitted to the following function:

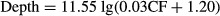


where Depth is the insertion depth into the ICC in mm and CF is the characteristic frequency in kHz. Among these identified single units, some also responded to INS, as shown as solid circles in Figure3. Interestingly, units in each animal that responded to INS were restricted to a small range along the path of the recording electrode. For example, in animal GP_Jul31, two units responding to INS were recorded. The CF range of the units for which INS evoked a response was 20.2–25 kHz. The corresponding depth into the ICC was 3.2–3.7 mm. In another animal GP_Sep24, six single units in the ICC responded to INS. The CF range of the units was 6–11.3 kHz. The corresponding depth was between 1.6 and 2.2 mm. Although the locations of activation in the ICC for each animal varied, all recordings corresponded to a narrow CF range roughly 0.6 octaves or 0.60 ± 0.09 mm at frequencies 6 kHz and above. Similar results were obtained for multichannel electrode recordings. Along the multichannel electrode array, usually only a limited number of adjacent channels (2–3) showed single unit activities responding to INS. These results clearly indicated a spatial selectivity in the ICC in response to INS and confirmed our previous results (Moreno et al. 2011; Richter et al. 2011). The recordings of neural responses obtained with multichannel electrodes were included in the group analysis and the depth of each active channel was estimated from the above depth–CF relationship (Figs.3, 4). For example, when the unit at the most distal channel had a CF at 20 kHz, the estimated depth was 2.95 mm. The depth of the units at other channels was then calculated accordingly. The frequency range covered by the length of the multichannel electrode (1.5 mm) is roughly 2.3 octaves, as calculated from the depth–CF equation. This frequency range is comparable to that estimated from ICC recordings with multichannel electrodes in other work (Snyder et al. 2004; Fig.4. Estimated frequency range: 3–18 kHz).

In total, 122 single units were identified in this study as shown in Table1. In hearing animals, the CF range of the recorded single units was between 0.5 and 25 kHz. This frequency range corresponded to a 0.05–3.60 mm insertion depth into the ICC, relative to its surface. The responses to acoustic and laser stimulations are summarized in Table1. The majority (62%, 64 of 103) of the single units from normal hearing animals responded only to acoustic stimulation. About 30% of the single units recorded in hearing animals responded to both acoustic and laser stimulation. About 8% of the single units in hearing animals only responded to laser stimulation. These laser-alone units were recorded in multiple normal hearing animals with both tungsten and multichannel electrodes at electrode insertion depths between 1.7 and 3.2 mm. The corresponding CF range was 5.8–20.0 kHz, which correlated with the location of the optical fiber along the cochlea. In such cases, the loss of responses to acoustic stimuli is likely attributed to the damage caused by the creation of cochleostomy, whereas direct activation of SGN by INS would account for the laser response of the single unit. Single unit activity evoked by INS was also recorded in both chronically deaf (14) and acutely deafened (12) animals. Surprisingly, none of these single units responded to any acoustic stimulation, although most of the ones recorded from acutely deafened animals responded to acoustic stimulation before the deafening. Acoustic evoked single units were also recorded occasionally in deaf animals.

**Table 1:** Summary of the single units responding to laser and acoustic stimulations

	Normal	Acute	Chronic
Total	103	12	16
Acoustic only	64	0	2
INS only	8	12	14
Acoustic & INS	31	0	0

Acoustic alone, responding to acoustic stimulation only; INS alone, responding to INS only; Acoustic & INS, responding to both.

### Higher limiting rate correlates with shorter latency

**Figure 5:**
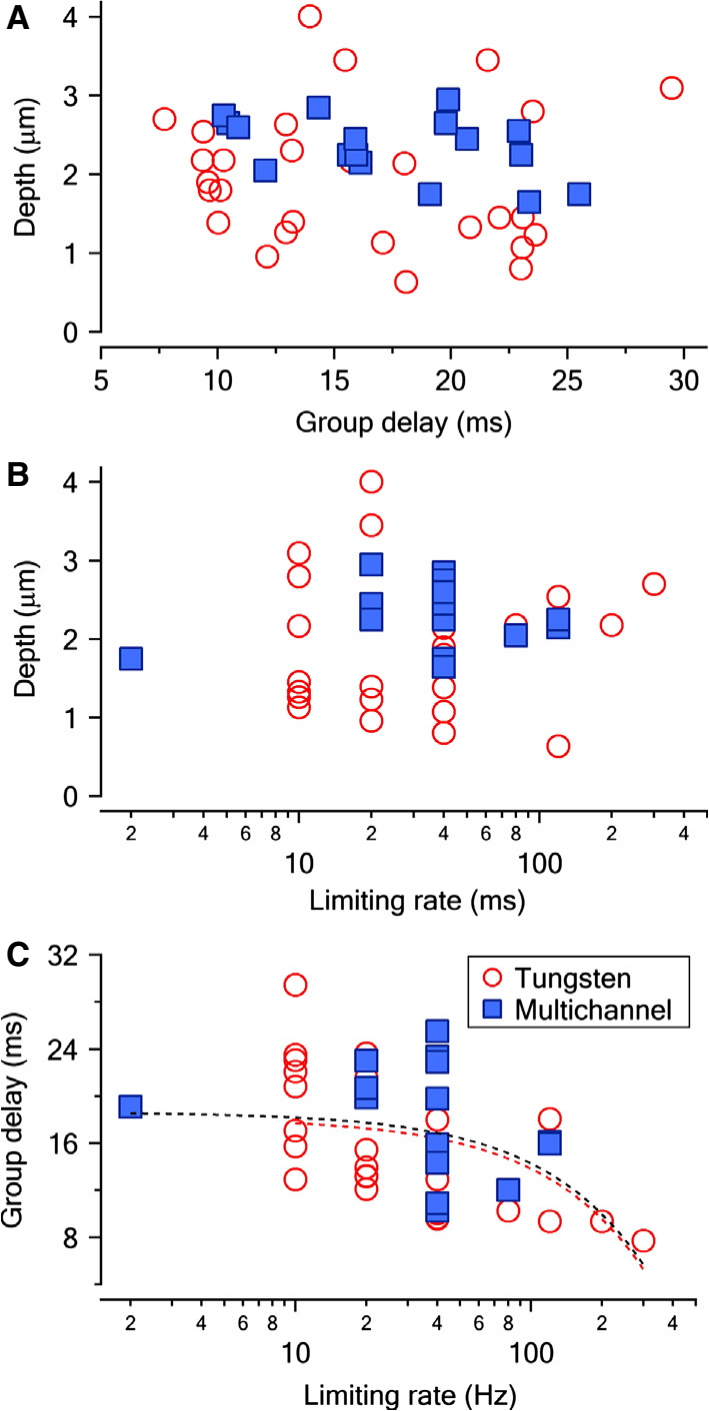
Pooled data showing the relationship between depth and group delay (A), depth and limiting rate (B) and group delay and limiting rate (C) for INS responses. The dashed lines are for the linear regression (red for tungsten single units and black for all the single units).

Fifty-six single units, which responded to INS, were obtained in this study. The group analysis is shown in Figures4 and 5. The distribution of the limiting rate was slightly different between the single units obtained with a single tungsten electrode (Fig.4A) and a multichannel electrode (Fig.4B). Results obtained with multichannel electrodes favored more single units, which had no phase-locked firing (one for tungsten and nine for multichannel) at any stimulation frequency. This difference is likely due to selection bias because for tungsten electrode recordings, units with better phase-locked responses were more likely to be selected for recording. Overall, most single units (28 of 31 which were recorded with tungsten electrodes and 23 of 25 recorded with multichannel electrodes) had limiting rates lower than 100 Hz. Results are shown as bar plots and as cumulative curves (Fig.4). The average limiting rate of all the units with phase-locked firing was 47.7 Hz and the highest limiting rate was 300 Hz.

To determine the correlation between the location, limiting rate, and group delay, corresponding scatter plots are shown (Fig.5). Neural responses to INS were recorded from neurons located 0.80–4.10 mm from the surface of the ICC. The distance was determined with the tungsten electrode as described in Materials and Methods section (Fig.5A and B). Multichannel electrodes did not represent the full frequency range of the guinea pig cochlea about 0.1–50 kHz (Heffner et al. 1971; Tsuji and Liberman 1997). The multichannel electrode was inserted 1.65–2.95 mm along the ICC, which correlated with the frequency range of 6.5–20 kHz (Fig.5A and B). The group delay varied between 7.71 and 29.46 msec with an average of 17.85 ± 5.72 msec (mean ± SD, Fig.5A and C). No significant correlation was found between the group delay and the depth (Fig.5A), or between the limiting rate and the depth (Fig.5B). However, a weak correlation between group delay and limiting rate was shown in Figure5C (*r*^2^ = 0.20). Higher limiting rates tended to associate with shorter group delays.

### Responses to acoustic stimulation and INS are different in ICC single units

With the goal in mind that INS replaces acoustic stimulation in the severely damaged cochlea, the response properties of the neurons should resemble each other for the two modes of stimulation. The following measurements quantify the differences between single unit response in the ICC during cochlear stimulation with acoustic clicks and with INS.

**Figure 6:**
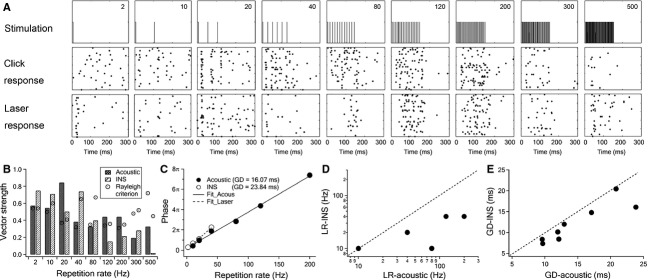
Comparison of the acoustic response and laser response in the same unit. (A) Raster plot of responses to click trains and INS. Panels in the 1st row indicate the stimulation waveform. Panels in the 2nd and 3rd row show the raster plots of click and laser responses, respectively. (B) Vector strength and Rayleigh criterion of the unit at different repetition rates. The vector strength values over the Rayleigh criterion are considered significant phase-locking to the stimulation. The limiting rates were 200 and 40 Hz for acoustic and laser stimulation, respectively. (C) Phase plots against repetition rate for both acoustic and laser responses in significant phase-locking ranges. Note that the group delays were different between the two stimulation paradigms. GD, group delay. (D and E) The plot of group delay and limiting rate, respectively, in response to acoustic and laser stimulations of a total of eight single units tested. Paired *t*-test showed statistically significant differences between acoustic and laser stimulations in both group delay (*P* = 0.02) and limiting rate (*P* = 0.01).

An example of a single unit is shown in Figure6. The raster plots revealed similar firing patterns to trains of laser pulses and trains of acoustic clicks (Fig.6A, rows 2 and 3). Although variations could be seen at low to middle repetition rates (2–80 Hz), the responses at higher repetition rates (over 120 Hz) were similar to each other, as seen in the raster plots. However, detailed analysis showed significant differences in terms of phase-locked firing in responses to acoustic and laser stimulations (Fig.6B). Reponses to INS showed a limiting rate at only 40 Hz, while responses to acoustic stimulation showed a much higher limiting rate of 200 Hz. Clearly the limiting rate to acoustic stimulation was much higher than that to INS in this unit. The phases of the responses to the two stimulation paradigms also showed linear relationships with repetition rates (Fig.6C). Group delay of the acoustic response calculated from the phase plot was 16.1 msec, much shorter than that of the laser response (23.8 msec).

In a total of eight single units tested with both, trains of laser pulses and acoustic clicks, responses to INS had lower limiting rates (paired *t*-test, *P *=* *0.012) and longer latencies (paired *t*-test, *P *=* *0.017) compared to those to acoustic stimulation (Fig.6D and E). These results show that the responses to INS and acoustic stimulation were different in ICC single units.

### The influence of deafening to the response properties of ICC neurons to INS

Although we demonstrated that INS could activate SGNs in deaf cochleae with little acoustic response, it was not determined whether there were contributions from hair cells. To address this question, we investigated the difference in ICC single unit responses pre- and postdeafening, as the majority of hair cells that were rendered nonfunctional through neomycin treatment (Leake-Jones et al. 1982) while SGNs were well preserved (Zappia and Altschuler 1989). Cochlear damage was verified by acoustic CAP recordings (Fig.1D) and the loss of responses to acoustic stimulation.

**Figure 7:**
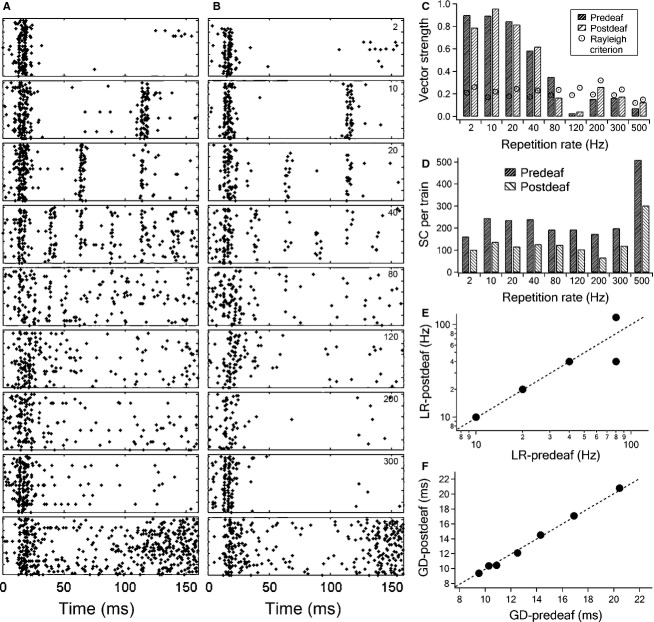
Comparison of the pre- and post-deafening responses in the same unit. (A and B) Raster plots of pre- (column A) and post-deafening (column B), respectively, in response to INS. The stimulation energy level was 142 *μ*J/pulse. Each row represents the response to one repetition rate (2, 10, 20, 40, 80, 120, 200, 300 and 500 Hz, respectively, ranging from top to bottom). (C) Comparison of pre- and post-deafening vector strength changes and Rayleigh criterion of the unit at different repetition rates. The limiting rate for pre- and post-deafening are 80 and 40 Hz, respectively. (D) Comparison of firing efficiency pre- and postdeafening at each repetition rate (SC: spike count) (E and F) The plot of pre- and post-deafening group delays and limiting rates, respectively, of a total of seven single units tested. Paired t-test showed no statistically significant differences between pre- and post-deafening in both group delay (*P* = 0.94) and limiting rate (*P* = 1). LR, limiting rate; GD, group delay.

Figure7 shows the pre- and postdeafening responses of an ICC single unit to INS recorded with a 16-channel electrode. Before deafening, the unit had a CF at 10 kHz and a high spontaneous firing rate of about 16 pulses/sec. The phase-locked firing turned into onset response at high repetition rates (Fig.7A). After deafening, the response to acoustic stimulation was lost (data not plotted), while the response to INS was similar to predeafening, as shown in raster plots. The spontaneous rate decreased to about 1.5 pulses/sec (Fig.7B). Detailed analysis showed that the vector strength varied similarly with different repetition rates pre- and postdeafening (Fig.7C). The limiting rate, however, was higher predeaf (80 Hz) than postdeafening (40 Hz). Nevertheless, this difference may be marginal and possibly secondary to the scarce firing at 80 Hz in the postdeafening animal, which is obvious in the raster plot (Fig.7B, panel 5). The group delay also remained the same pre- and postdeafening (10.3 and 10.4 msec). The firing efficiency of the unit was almost identical in both pre- and postdeafening, although predeafening spike counts were higher than those after deafening (Fig.7D). Comparison of six other single units showed that the response properties also kept consistent pre- and postdeafening (Fig.7E and F). Although the changes in temporal properties pre- and postdeafening in these units were not significant, we did observe the loss of INS responses after deafening in some units.

## Discussion

The results demonstrated that INS evoked single unit responses for a narrow range along a track into the ICC in both normal hearing and deaf animals. The spatial selectivity of ICC activation in the present study is consistent with the previous report (Richter et al. 2011). Neuronal responses recorded with single tungsten or multichannel electrodes have similar temporal properties except the location difference. The ICC spatial selectivity of INS is comparable to that obtained with acoustic stimulation (Richter et al. 2011) or the bipolar configuration of electrical stimulation (Snyder et al. 2004; Middlebrooks 2008). The average activation range of INS in the ICC was roughly 0.6 octaves or 0.6 mm, comparable to that evoked in auditory fibers (Richter et al. 2011). The responses of ICC neurons to INS showed lower limiting rates and longer latencies when compared to those for acoustic stimulation. The temporal properties and responses of ICC neurons to INS in normal hearing animals were similar before and after acute deafening. Overall, these results all indicate that the temporal properties of ICC neurons in response to INS are different from those to acoustic stimuli.

### Temporal properties of neural recordings from ICC single units during INS

We reported previously that the average discharge rate of single auditory nerve fibers in response to INS was about 100 Hz and the limiting rate was usually less than 100 Hz (Littlefield et al. 2010). In this study, we showed that the ICC single units held similar temporal properties as those of the auditory nerve. The results demonstrate that the average limiting rate of the ICC single units was only close to 50 Hz. This low limiting rate is typical in ICC neurons in response to acoustic signals. It is not clear whether the low limiting rate of the ICC single units is predicative on the ability to encode high frequency signals. For representing the fine structure of the acoustic signal, this is seemingly insufficient. On the other hand, one can argue that large populations of (independently firing) neurons can achieve the coding and represent the fine structure. In our recordings, multiple single units with different waveform features and/or temporal properties were recorded in response to INS at the same cochlear site. This suggests that a group of neurons could be activated and achieved fine structure coding with a population of neurons.

### Factors shaping the temporal properties of ICC neurons in response to INS

In contemporary cochlear implants, the temporal structure of acoustic stimulation is encoded by amplitude modulated pulse trains at carrier repetition rates of typically 250–1800 Hz (Vandali et al. 2000; Arora et al. 2011). Yet, consensus has not been reached on whether higher repetition rates are better in temporal information transmission (Rubinstein et al. 1999; Vandali et al. 2000; Middlebrooks 2008; Arora et al. 2011). In response to INS, most ICC units have phase-locked firing at repetition rates only up to 80 Hz (Fig.4). The limiting rate of ICC neurons to INS is about half the values for electrical stimulation (Snyder et al. 1995; Vollmer et al. 1999; Middlebrooks and Snyder 2010). The neurons responding to both INS and acoustic stimulation have a limiting rate approximate four times lower in response to INS than to click trains (Fig.7). Furthermore, the mean group delay in response to INS is about 1.2 times longer than that of acoustic stimulation, as shown in Figure7E and in accordance with other studies (Syka et al. 2000).

Improved spatial selectivity of INS in the cochlea may be able to explain the response property of ICC neurons. It is expected that spatially more selective activation of SGNs would elicit less synchronous input in brainstem nuclei such as dorsal and ventral cochlear nuclei, superior olivary complex and lateral lemniscus. Previous studies showed that ICC neurons receive projections from multiple sources, including ascending nuclei in brainstem (Semple and Aitkin 1981; Maffi and Aitkin 1987). Temporal properties of ICC neurons thus can be shaped by these convergent innervations, and less synchronous inputs lead to lower temporal fidelity shown as lower limiting rate and lower firing efficiency. Similarly, longer latency is also needed to integrate excitatory inputs to ICC neurons in INS and reach firing threshold. Similar observations were made in a study comparing monopolar and bipolar electrode configurations using electrical stimulation (Middlebrooks 2008), in which the bipolar configuration with a narrower activation range was associated with lower temporal fidelity in auditory cortex neurons. Another report in support of this hypothesis is that, improved spatial selectivity of INS results in a much narrower spatial tuning curve in the ICC (Richter et al. 2011) compared to acoustic and monopolar electrical stimulation.

In addition, location bias of INS might also contribute to the temporal properties of ICC neurons in this study, as was shown in previous studies (Snyder et al. 1990, 1991; Leake et al. 1991). High limiting rate neurons may originate at the apex of the cochlea, which are characterized by low CFs, short latencies, and are thought to play a role in the transmission of temporal fine structure of the stimulation (Middlebrooks and Snyder 2010), which might be limited in our results (Fig.5). Nevertheless, further studies are still needed to determine other factors shaping the temporal properties of ICC neurons to INS, as well as to clarify whether high temporal fidelity in terms of limiting rate is necessary for signal transmission. For example, INS in the middle or apical part of the cochlea might explain location specific contributions to ICC neurons.

### Challenges for cochlear INS

Heating of the target volume for INS results in the expansion of the volume and subsequent stress relaxation or pressure waves, which may mechanically vibrate the basilar membrane. Since the deafening procedure in our experiments does not necessarily remove all hair cells, a direct stimulation of hair cells, as has been described for the vestibular system, cannot be ruled out (Rajguru et al. 2011). Evidence for such behavior comes from experiments conducted in the guinea pig (Schultz et al. 2012; Baumhoff et al. 2013; Thompson et al. 2015) and rats (Verma et al. 2014). The responses to INS were always correlated with residual cochlear function. Laser-induced mechanical vibration of the basilar membrane was also demonstrated in a recent study (Ren et al. 2014). A passive movement and an active travelling wave of the basilar membrane were generated by direct laser irradiation of the basilar membrane in pristine cochlea. However, this laser-evoked active process underlying cochlear amplification does not happen in damaged cochleae (Ren et al. 2014; and personal communications with Dr. Ren). The present experiments do not support a general stimulation of the cochlea by a stress relaxation wave. On the contrary, the response to INS in damaged cochlea, the different response properties to INS and acoustic stimulation in the same neuron, and the similarity before and after deafening, all indicated a direct stimulation of SGNs. The role of the optoacoustic events on hair cells is not dominant in our study. Even for hearing animals, only units that respond acoustically to a limited frequency range of less than an octave can be stimulated with the laser. In addition to that, for some hearing animals, neurons were identified that only responded to INS and not to acoustic stimulation (Table1). In cochleae from normal hearing animals all the SGNs should be activated by acoustic stimulation. A reasonable explanation for the units in the ICC that were only stimulated by the laser is that hair cell loss caused by the cochleostomy led to the lack of acoustic response and INS directly excited spiral ganglion neurons. The depth and estimated frequency range of these units further supported this view (Fig.3).

Improved spatial selectivity outperforms electrical stimulation in terms of possible independent channels. The application of INS requires the capability of direct stimulation of SGNs, which makes INS-based prosthesis mechanically feasible. Since the deafening procedure in our experiments does not necessarily remove all hair cells, a direct stimulation of hair cells, as has been described for the vestibular system, cannot be ruled out (Rajguru et al. 2011). Further detailed experiments in an animal model that truly lacks hair cells are required to resolve the open question.

In summary, cochlear INS shows a spatial selectivity in ICC and the temporal properties of ICC neurons show lower limiting rate, longer latency, and lower firing efficiency. INS is also able to evoke ICC neuronal activities in acute or chronically deaf animals. Further studies are needed to better determine the involvement of remaining hair cells in INS in deaf animals.

## Conflict of Interests

None declared.
